# Prevalence of anxiety in patients with an implantable cardioverter defibrillator: measurement equivalence of the HADS-A and the STAI-S

**DOI:** 10.1007/s11136-019-02237-2

**Published:** 2019-06-22

**Authors:** W. H. Emons, M. Habibović, S. S. Pedersen

**Affiliations:** 1 Department of Methodology and Statistics, Tilburg University, Tilburg, The Netherlands; 2 Department of Medical and Clinical Psychology, Tilburg University, Tilburg, The Netherlands; 3 Department of Cardiology, Elisabeth-TweeSteden Hospital, Tilburg, The Netherlands; 4 Department of Psychology, University of Southern Denmark, Odense, Denmark; 5 Department of Cardiology, Odense University Hospital, Odense, Denmark

**Keywords:** Anxiety, Implantable cardioverter defibrillator, Assessment, Screening

## Abstract

**Purpose:**

The Hospital Anxiety and Depression Scale (HADS-A) and State-Trait Anxiety Inventory (STAI-S) are popular instruments for assessing anxiety and are considered interchangeable, although little is known about their equivalence. Hence, we examined whether the two instruments are (i) equivalent with respect to determining the prevalence of probable clinical anxiety levels and (ii) reflect variation on a common anxiety attribute.

**Methods:**

Score and construct concordance were evaluated using equipercentile equating and bifactor modeling, respectively. Secondary data from the WEBCARE trial and the MIDAS study were used for the current study, where patients implanted with a first-time implantable cardioverter defibrillator completed both the HADS-A and the STAI-S within 10 days post implant.

**Results:**

Data from 710 patients were included in the analyses. Results showed that the STAI-S produced a higher prevalence rate than the HADS-A (39% vs. 23%). A crosswalk table was generated with equivalent scores and cutoffs for the HADS-A and STAI-S, respectively. Bifactoring suggested that HADS-A and STAI-S largely tapped into the same generic anxiety attributes.

**Conclusions:**

STAI-S and HADS-A reflect a common anxiety attribute, but using the recommended cutoff scores on the respective measures show very different prevalence rates and would classify patients as anxious with the STAI-S who would not be identified as such with the HADS-A. Clinicians and researchers should be aware of the inequivalence when using these measures for screening and determining the prevalence of probable clinical anxiety levels.

## Introduction

The implantable cardioverter defibrillator (ICD) is the first line of treatment for life-threatening ventricular tachyarrhythmias (VTa’s) both as primary (patients who have an increased risk of experiencing VTa’s) and secondary prophylaxis (patients who have previously experienced VTa’s) [1–3]. Although the ICD is well accepted by the majority of patients [4], one in four patients experiences psychological distress post implantation, including anxiety and depression [5]. Distress has not only been associated with the development of posttraumatic stress disorder post implantation [6–8], but on its own or in combination with the distressed (Type D) personality profile also with increased risk for VTa’s and even mortality [9, 10].

In order to identify ICD patients who suffer from anxiety and depression, it is paramount that we have appropriate and well-validated measures available with appropriate cutoffs and sensitivity and specificity. Generally, a distinction is made between generic and disease-specific anxiety measures [11]. Examples of generic measures include the Hospital Anxiety and Depression Scale (HADS) [12], the Spielberger Trait-State Anxiety Inventory (STAI) [13], the Beck Anxiety Inventory (BAI) [14], and the Generalized Anxiety Disorder Scale (GAD-7) [15]. Recently, disease-specific measures were also developed, such as the Florida Shock Anxiety Scale (FSAS) [16, 17] and the ICD Patient Concerns questionnaire (ICDC) [18] that tap into anxiety related to ICD shocks.

Since ICD-related anxiety seems to be more prevalent than shock anxiety [18], this study focused on two widely used generic anxiety scales, the (state) anxiety scales from the HADS and the STAI, referred to as the HADS-A and STAI-S, respectively. Both instruments are generic and commonly used for screening for anxiety in the general population, psychiatric and somatic patients, including cardiac patients and ICD patients. Although they are considered interchangeable, we know little about their equivalence in terms of identifying patients with probable clinical anxiety levels and whether they both reflect a common anxiety attribute. To our knowledge, no information is available whether the cutoffs used for the HADS-A and the STAI-S reflect comparable anxiety levels and thus produce equivalent prevalence rates. Should that not be the case, the choice for either the HADS-A or STAI-S may have consequences for treatment and care if patients are identified with anxiety on one scale but not the other and also for the results of epidemiologic research. Ideally, prevalence rates and treatment decisions should be independent of the specific anxiety measure used. Moreover, the HADS-A and STAI-S are supposed to reflect a common psychological attribute of *anxiety*, but they may tap into other psychological subdomains as well, as their items are framed differently. Previous research has shown that in addition to anxiety, the HADS-A also taps into relaxed affect [19]. The STAI-S on the other hand measures anxiety in terms of the presence versus absence of symptoms, which appear to be separate factors, as determined by factor analysis [20]. Hence, subtle differences in the meaning of HADS-A and STAI-S scores may explain prevalence differences and the power to predict adverse treatment outcomes.

Hence, in the current study, we examined whether the HADS-A and STAI-S (i) are equivalent with respect to determining the prevalence of anxiety symptomatology as reflected in a crosswalk table for equivalent scores, (ii) reflect variation on a common anxiety attribute in ICD patients.

## Methods

### Data collection design and participants

Secondary data analyses were performed using data from the following two studies: Mood and personality as precipitants of arrhythmia in patients with an implantable cardioverter defibrillator: a prospective study (MIDAS) cohort [21, 22] and the WEB-based distress management program for implantable CARdioverter dEfibrillator (WEBCARE) trial [23]. Each respondent completed both questionnaires (HADS-A and STAI-S) within 2 weeks post ICD implantation. The total sample comprised 788 participants. For 74 (9.4%) participants, data on either the HADS-A or STAI-S were (almost) completely missing and four participants (0.4%) had three to seven missing values. These participants were removed from the analyses. For the remaining participants, missing scores were imputed for each scale separately by means of two-way imputation [24, 25], resulting in a data set of *N* = 710 complete cases. Table 1 shows the sample characteristics of this study sample. For more information about background sample characteristics of the original studies, the reader is referred to previous publications of the MIDAS and WEBCARE study [22, 23].

**Table 1:** Background characteristics and descriptive statistics for HADS-A and STAI-A scores for the current study sample (*N* = 710)

Background variable	Total	Per database	
		WEBCARE	MIDAS
	*N* = 710	*n* = 287	*n* = 423
Gender			
%males	79.2	79.4	79.0
Age (years)			
Mean	58.53	58.94	58.25
SD	11.11	10.3	11.6
Median	60	61	59
25th and 75th quartiles	52–67	53–67	52–67
Min; max	17; 81	19; 79	17; 81
ICD indication			
%primary	66.3	68.9^a^	64.5
Charlson’s comorbidity index (CCI)			
Mean	1.93	1.67^b^	2.08^c^
SD	1.58	1.04	1.8
Median	1	1	1
25th; 75th quartiles	1; 3	1; 2	1; 3
Min; max	0; 7	0; 6	0; 7
Beta blockers			
%yes	80.8	82.2^a^	79.9
ACE-inhibitors			
%yes	67.5	61.2^a^	71.9
NYHA-class			
%New York Heart Association classification	27.3	21.2^d^	30.6^e^
LVEF			
%left ventricle ejection fraction < 35%	82.3	76.1^f^	86.4^g^
Heart failure			
%yes	44.9	54.2^a^	38.5
HADS-A (total scores)			
Mean	5.13	4.60	5.48
SD	3.72	3.28	3.95
Reliability (Cronbach’s alpha)	.83	.84	.83
SEM	1.51	1.31	1.62
STAI-S (total scores)			
Mean	37.23	35.25	38.57
SD	11.46	10.33	11.89
Reliability (Cronbach’s alpha)	.95	.94	.95
SEM	2.62	2.59	2.63

^a^*n* = 286, ^b^*n* = 240, ^c^*n* = 417, ^d^*n* = 231, ^e^*n* = 421, ^f^*n* = 243, ^g^*n* = 361

### Measures

#### HADS anxiety scale

The HADS-A is one of the two subscales that forms the HADS [12]. The scale comprises seven items. For each item, respondents have to mark one of four response options from 0 to 3, with a higher score indicating a higher level of anxiety. Five items are positively worded (e.g., ‘I feel anxious’), and two are negatively worded (e.g., ‘I feel at ease’). The sum score ranges from 0 to 21. Scores of 8 or higher define clinical levels of anxiety, although other diagnostic cutoffs have been recommended as well (e.g., [26]). The dimensionality of the HADS has been extensively studied [27]. These studies consistently found the presence of a restlessness factor, which comprises two items from the anxiety scale and one from the depression scale. The HADS and its subscales have shown to have good psychometric properties; for the HADS-A subscale, the internal consistency as measured by Cronbach’s alpha was 0.82 in the WEBCARE cohort and 0.83 in the MIDAS cohort.

#### Spielberger’s State-Trait Anxiety Inventory (STAI-S)

The STAI-S of the Spielberger’s State-Trait Anxiety Inventory comprises 20 items. Items are answered on a 4-point Likert scale ranging from 1 (*not at all*) to 4 (*very much so*), with the total score ranging from 20 to 80, with higher scores indicating higher levels of anxiety symptoms. A cutoff score of 40 is commonly used to define probable clinical levels of anxiety. The STAI-S has shown to be a valid and reliable measure. The internal consistency, as measured by Cronbach’s alpha ranged from .94 to .95 in the WEBCARE and MIDAS cohorts. Using factor analysis, Vigneau and Cormier [20] showed that the indicative items of anxiety (e.g., ‘I am worried’ and ‘I feel nervous’) and the contra-indicative items (e.g., ‘I feel calm’ and ‘I feel pleasant’) form two different correlated dimensions.

### Statistical analysis

The first objective of this study was to define a crosswalk table between scores from the HADS-A and the STAI-S. Using this crosswalk table, clinicians can convert the scores obtained on the HADS-A into comparable scores on the STAI-S, and vice versa. This allows clinicians to compare anxiety levels between patients even though the scores were obtained from different measures. To find the crosswalk, we used a psychometric approach known as equipercentile equating [28, 29]. Within this framework, scores on two different tests are assumed to reflect comparable attribute levels if they have the same percentile rank in the population. For example, if we know that 10% of the persons in the population scores 3 or less on the HADS, then we can look for which score on the STAI-S it also holds that 10% of the people have that score or lower. Suppose this is a score of 11. In that case, a score of 3 on the HADS-A is considered to reflect similar anxiety levels as a score 11 on the STAI-S. Because the HADS-A and STAI-S were developed according to different specifications, one speaks of a concordance relationship between scores [30, 31] and this relationship is summarized in the crosswalk table.

Because all respondents completed both test forms, crosswalk tables can easily found by matching scores from different tests that have corresponding percentile ranks [30]. However, to find the percentile ranks, we use the distribution of test scores in the sample, which may have strong irregularities due to sampling errors. There irregularities are specific for the sample at hand, and results based on it may have limited the generalizability. To alleviate the problem, one may smooth out irregularities in the distribution. These distributions are less sensitive to sampling errors. To accomplish this goal, we used log-linear modeling, which results in smoothed distributions that have the same mean, SD, skewness, and kurtosis as the observed scores in the sample, but without irrelevant irregularities [29]. Because a detailed technical explanation of this method would be rather lengthy and beyond the scope of this paper, we only discussed the main points refer the reader to Von Davier et al. [29] for further technical details. All computations were done using the R package kequate [32]. R-code can be obtained upon request from the first author.

#### Research objective 2: construct concordance

**Fig. 1:**
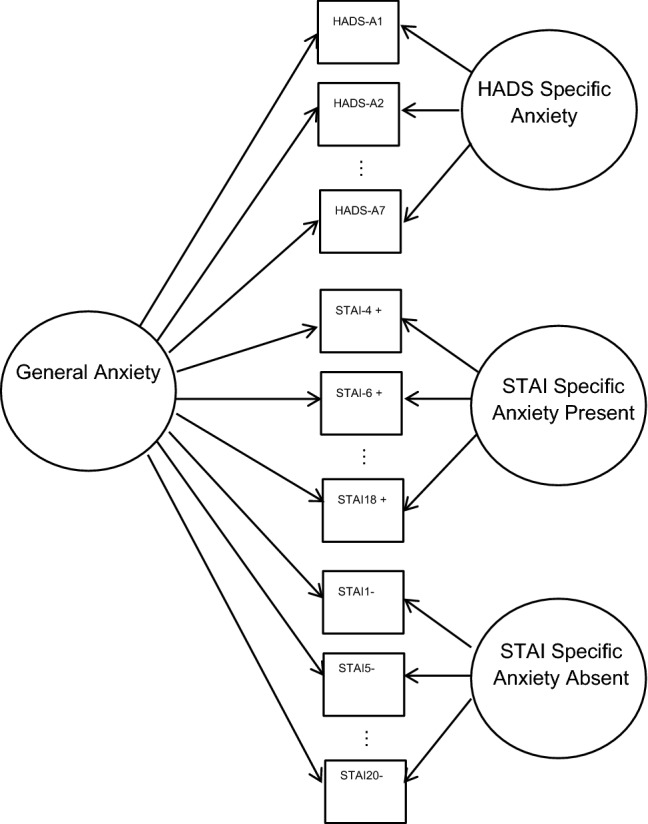
Graphical representation of the bifactor model

To explore to what extent a single generic state-anxiety attribute can explain the responses to both the HADS-A and the STAI-S, we fitted a bifactor model (e.g., [33, 34]; see Fig. 1). The postulated bifactor model included a general factor on which all items load, and three specific factors: one specific factor on which only HADS items load; one on which only STAI-S present (positively worded) items load; and one on which only STAI-S absent (negatively worded) items load. The choice of two specific factors for the STAI-S was based on Vigneau and Cormier [20]. Conceptually, the postulated bifactor model assumes that the association between the HADS-A and STAI-S items is explained by their dependence on a common general anxiety attribute. The specific factors explain associations between the HADS-A items, the STAI-S present items, or STAI-S absent items and thus represent scale-specific differences in anxiety. The (standardized) general loadings in the bifactor model show the extent to which the items tap into the same underlying construct. The loadings on the specific factors show to what extent the item represents unique scale-specific variance [34]. For generic measures, ideally the loadings on the general factor are large and on the specific factor low. All models were fitted on the polychoric correlation matrix using MPLUS-5 [35] employing the ULSMV estimator (MPLUS syntax available upon request).

## Results

### Sample characteristics

The complete cases of the MIDAS cohort comprised 79% males and 21% females. The mean age of the MIDAS sample was 58.4 (SD 12.2). Males were on average older than females (mean ages were 54.0 and 59.5 for females and males, respectively; difference is significant at the 5% level, *t*(421) = 3.823, *p* < .001). The complete WEBCARE sample comprised 81.2% males and 18.8% females. Mean age was 58.36 (SD 10.016), with a mean age of 59.6 for males and 53.2 for females (difference is significant at the 5% level, *t*(285) = 4.309, *p* < .001). Sample characteristics of the cases with missing data did not systematically differ from the complete cases.

Table 1 shows the descriptive statistics, the internal consistency (coefficient alpha), and standard error of measurement of the total scores. Even though both the MIDAS and the WEBCARE cohorts represent the same cardiac population of interest, patients from the MIDAS cohort were on average more anxious than those in the WEBCARE cohort at the time of measurement (Cohen’s *d* = 0.23 for the HADS-A, and *d* = 0.30 for the STAI-S; differences were significant at the 5% level). The correlation between HADS-A and STAI-S total scores was 0.74. Table 2 shows the prevalence rates of probable clinical anxiety using the conventional clinical cutoffs for the HADS-A and STAI-S, both for the complete sample and for each sample separately. The prevalence rate was higher for the STAI-S than for HADS-A. Differences were significant at the *p* < .01 significance level when tested with the McNemar test [36]. Hence, results suggest that the STAI-S uses a more liberal cutoff for diagnosing probable clinical anxiety than the HADS-A.

**Table 2:** Observed prevalence rates (percentages) of clinical anxiety for the HADS-A and the STAI-S using conventional cutoff scores

Sample	Prevalence in probable clinical anxiety		Difference (%)
	HADS-A (%)	STAI-S (%)	
WEBCARE	18	30	12*
MIDAS	27	45	18*
Total sample	23	39	16*

*p < 0.001 (McNemar test)

### Results for research objective 1: score concordance

**Fig. 2:**
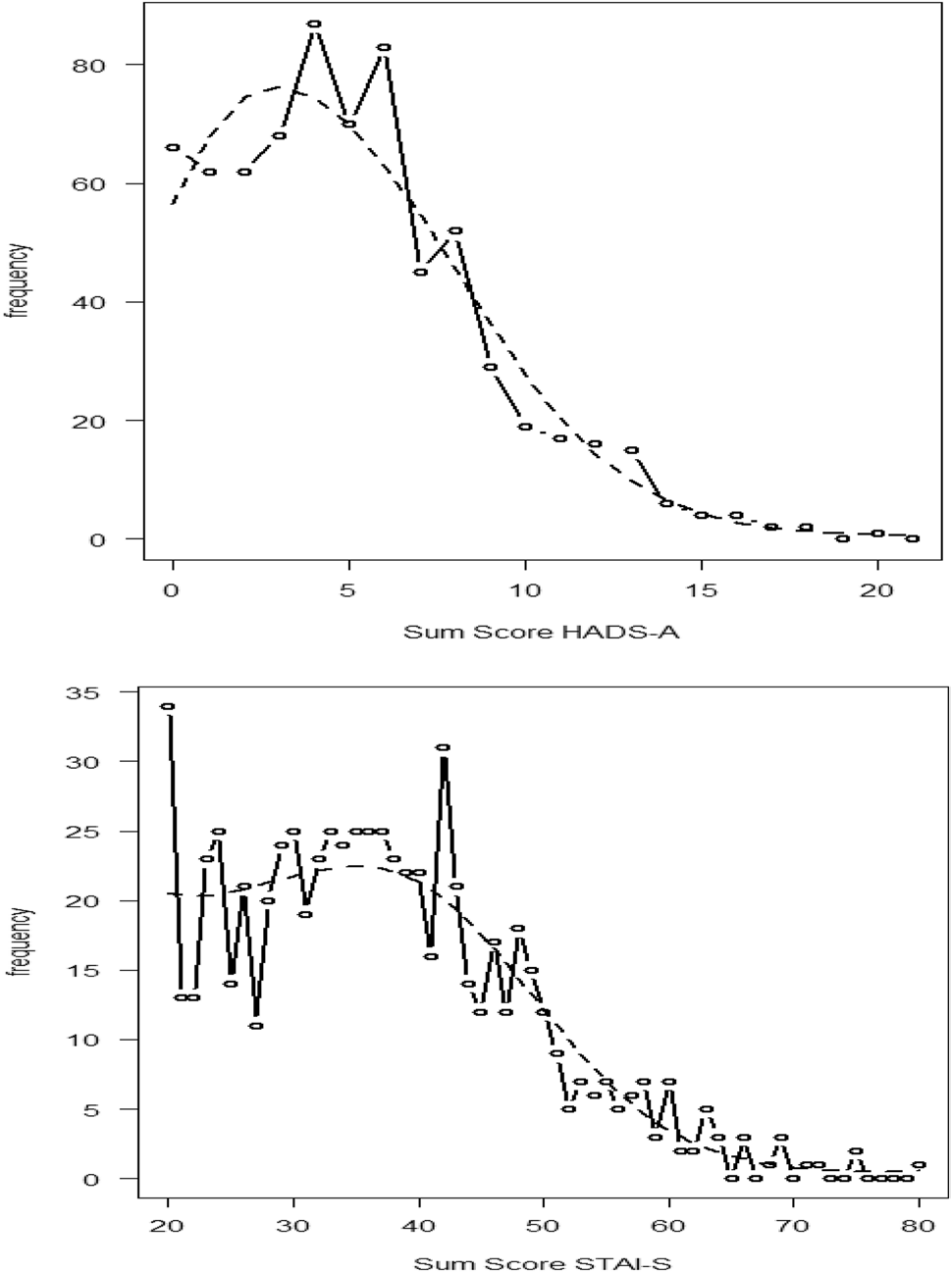
Observed frequency distribution raw scores for the HADS-A and the STAI-S

Figure 2 shows the observed (dotted line) and smoothed (dashed line) frequency distributions of the total scores for both scales. The resulting concordance relationship resulting from the smoothed frequencies is shown in Table 3, which is a so-called crosswalk table. Because the raw score scales for the HADS-A and STAI-S differ in the number of score points, the scores from the HADS-A correspond with a range of scores on the STAI-S and vice versa. For example, a score of 6 on the HADS-A is concordant with scores in the range of 40–42 on the STAI-S. Likewise, a score in the range of 51–53 on the STAI-S is concordant with a score of 10 on the HADS-A.

**Table 3:** Crosswalk table for the HADS-A and STAI-S

HADS-A	STAI-S
0	20–22
1	23–25
2	26–29
3	30–32
4	33–35
5	36–39
6	40–42
7	43–44
8	45–47
9	48–50
10	51–53
11	54–56
12	57–58
13	59–61
14	62–64
15	65–68
16	69–71
17	72–74
18	75–76
19	77–78
20	79
21	80

Concordant clinical cutoffs were obtained using linear interpolation (e.g., [29]). First, consider the cutoff of 8 for the HADS-A. In particular, a score of 8 on the HADS-A corresponds to a score of 46.46 on the STAI-S. Rounding the value to the nearest integer suggests a cutoff of 46. The STAI-S equivalent cutoff for the HADS-A can be obtained in the same way. The conventional clinical cutoff of 40 on the STAI-S corresponds with an interpolated cutoff score of 5.82 on the HADS-A. Thus, the rounded cutoff on the HADS-A, which corresponds to the conventional clinical cutoff on the STAI-S, equals 6 and is two points lower than the conventional cutoff.

**Fig. 3:**
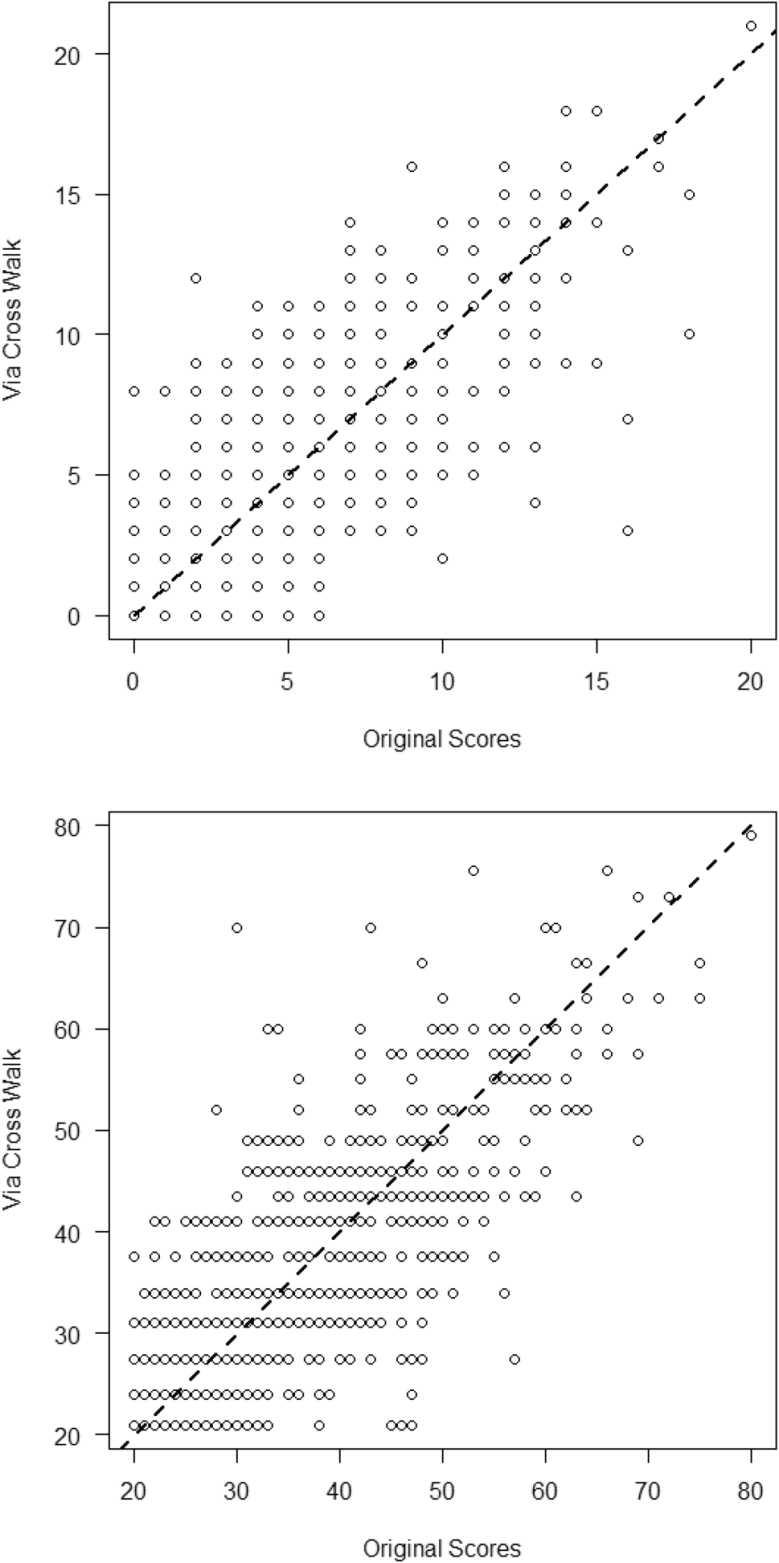
Scatter plots

To evaluate the accuracy and generalizability of the concordance relationship as shown in Table 3, for each scale, we compared the actual scores with the linked scores that would be obtained via the score on the other test (henceforth referred to as crosswalk scores). For example, the crosswalk HADS score for a STAI-S score of 50 equals 9. Figure 3 shows the scatter plots of original scores against the crosswalk-based scores, which shows no evidence of systematic bias because observations were scattered around the identity line. Correlations between the actual and crosswalk scores were 0.75, both for the HADS-S and STAI-A. Table 4 shows the crosswalk table of the clinical classifications using the actual scores and crosswalk scores. In particular, columns 3 and 4 show the proportions of inconsistent classifications (i.e., being classified in the diagnostic category by the actual score and in the non-diagnostic category by the pseudoscore, or vice versa). Using HADS-A concordant cutoffs (upper panel), the total proportion of inconsistent classifications ranged from 0.15 (WEBCARE and total sample) to 0.19 (MIDAS cohort). Results were less favorable using STAI-S concordant cutoffs (lower panel), where the proportion of inconsistent classifications ranged from 0.22 (MIDAS and total sample) to 0.23 (WEBCARE). According to Koch and Landis’ [37] rules of thumb for Kappa (*κ*), decisions based on actual and pseudoscores were in moderate agreement. Columns 8 and 9 show the overall prevalence rates. Except for the WEBCARE cohort and STAI-S equivalent cutoffs, these rates are close to each other. This agreement in overall prevalence rates stems from using equipercentile equating for determining concordant diagnostic cutoffs.

**Table 4:** Classification consistency and prevalence rates of clinical anxiety for the HADS-A and STAI-S equivalent cutoff scores

Sample	Cross classification^a^					Prevalence	
	H−/S−	H−/S+	H+/S−	H+/S+	*κ*	HADS-A	STAI-S
Using HADS—equivalent cutoff for the STAI-S							
WEBCARE	0.75	0.07	0.08	0.10	0.52	0.18	0.18
MIDAS	0.64	0.09	0.10	0.17	0.52	0.27	0.26
Total sample	0.68	0.08	0.09	0.15	0.52	0.24	0.23
Using STAI—equivalent cutoff for the HADS-A							
WEBCARE	0.54	0.07	0.16	0.23	0.54	0.39	0.30
MIDAS	0.45	0.12	0.10	0.34	0.54	0.43	0.45
Total sample	0.49	0.10	0.12	0.29	0.54	0.42	0.39

The upper panel gives the classifications using the conventional cutoff of 8 for the HADS-A and the concordant cutoff of 46 on the STAI-S. The lower panel gives the classification proportions using the conventional cutoff of 40 for the STAI-S and the concordant cutoff of 6 on the HADS-A

^a^H− score below cutoff on the HADS-A, H+ score at or above the cutoff on the HADS-A, S− score below cutoff on the STAI-S, S+ score at or above the cutoff on the STAI-S

### Results for research objective 2: construct concordance

Table 5 (Columns 2 to 5) shows the standardized loadings in the bifactor model. Because for some items the specific-factor loadings were non-significant at the two-tailed 5% level, results are presented for the bifactor model in which non-significant loadings were fixed to 0. This means that some of the items only load on the general factor. Fit indices showed adequate fit; RMSEA was 0.062, TLI was 0.99, and CFI was 0.94. Residual correlations had a mean of 0, an SD of 0.037, and ranged from − 0.091 to 0.174. About 2.3% of the residual correlations were larger than 0.10. Results suggest that the fit of the bifactor model is acceptable given the purpose envisaged. We also fitted the 1-factor model, but the fit indices indicated poor fit of the model. The bifactor model was therefore retained for further analysis.

**Table 5:** Standardized loadings under the one-factor model and bifactor model

	Bifactor model			
	General factor	HADS-A	STAI-S absent	STAI-S present
HADS-A 1	0.79	0.45		
HADS-A 2	0.71	0.35		
HADS-A 3	0.81	0.46		
HADS-A 4	0.82	0.32		
HADS-A 5	0.73	0.31		
HADS-A 6	0.71	0.13		
HADS-A 7	0.77	0.29		
S-STAI1–	0.65		0.22	
S-STAI2–	0.78		0.31	
S-STAI3+	0.65			0.25
S-STAI4+	0.72			0.23
S-STAI5–	0.78		0.33	
S-STAI6+	0.79			0.00^a^
S-STAI7+	0.56			0.00^a^
S-STAI8–	0.77		0.54	
S-STAI9+	0.65			0.10
S-STAI10–	0.80		0.55	
S-STAI11–	0.72		0.49	
S-STAI12+	0.65			0.58
S-STAI13+	0.72			0.52
S-STAI14+	0.75			0.00^a^
S-STAI15–	0.71		0.38	
S-STAI16–	0.74		0.57	
S-STAI17+	0.61			0.00^a^
S-STAI18+	0.58			0.18
S-STAI19–	0.57		0.41	
S-STAI20–	0.64		0.55	

	Explained total variance			
HADS-A	43%	12%		
STAI-S	53%		10%	4%
Total	51%	3%	7%	3%

	Explained common variance (ECV)			
HADS-A	78%	22%		
STAI-S	79%		15%	6%
Total	79%	5%	12%	4%

Standardized loadings were obtained using Parallel Axis Factoring (ULSMV) estimation in MPLUS

^a^Loadings were restricted to zero

Inspection of the general-factor loadings showed that all items, both from the HADS-A and STAI-S, showed considerable standardized loadings (≥ 0.56) on the general factor. Interestingly, the loadings on the general factor in the bifactor model and those in the one-factor model were not appreciably different; the largest difference was 0.09. Thus, the factor from the one-factor model has the same meaning as the general factor in the bifactor model. The general factor in the bifactor model explained 43% of the total variance of the HADS-A, 53% of the total variance of the STAI-S items, and 51% taking all items together. The specific factors of the STAI-S accounted for 10% (anxiety absent) and 4% (anxiety present) of the variance, and the specific factor of the HADS-A for 12%. Hence, the general factor accounts for 78% to 79% of the common variance (i.e., explained common variance). Also computed were the reliability coefficients for measuring the general factor (coefficient Omega-h; e.g. [33]). Reliabilities were 0.71 for the HADS-A, 0.88 for the STAI-S, and 0.91 for the complete item set; thus, all above commonly accepted standards. These results suggest that there is a general factor of state anxiety that is predominant and this general factor can be fairly reliably measured using the HADS-A, reliably by the STAI-S, and most reliably when the scales are combined.

## Discussion

Ideally, cutoffs of different anxiety scales should reflect comparable levels of anxiety, but this study shows that the cutoffs of two widely scales, the HADS-A and STAI-S, do not (necessarily) yield similar screening criteria even though they are based on external criteria. In particular, the diagnostic cutoff on the STAI-S corresponding to the cutoff on the HADS-A is about six points higher than the conventionally used cutoff of 40 for the STAI-S. Likewise, the concordant cutoff of the STAI-S on the HADS-A is two points lower than the conventional cutoff. This means that using the ‘traditional’ cutoff scores for anxiety the STAI-S would classify patients as anxious who would not be identified as such by the HADS-A. Hence, the STAI-S appears to be less conservative and would thus produce a higher prevalence rate compared to HADS-A. Using the crosswalk table, equivalent cutoffs for different scales can be employed so as to obtain prevalence rates that are less sensitive to the scale that is used.

Our findings further suggest that HADS-A and STAI-S largely tap into the same generic anxiety attribute, although both scales also have their unique parts. Hence, results confirm that both questionnaires reflect somewhat different operationalization of state anxiety. However, if it is the common generic anxiety attribute that explains adverse health outcomes in ICD patients, both scales may be feasible, but results also showed that HADS-A measurements of the general anxiety trait were less precise than STAI-S. Future research should focus on the relationship between general and specific trait variation and the prognostic and screening properties of both questionnaires.

Both questionnaires are widely used as screening tools in research and clinical practice. They have shown to be predictive of adverse health outcomes in cardiac populations [23] but have also received some critique over the past years. For example, it has been advocated that the HADS should be abandoned due to an unclear latent structure [38]. However, others have proposed that the scale should at best be restructured [39], as it is a strong predictor of morbidity and mortality in cardiac patients regardless of its structure [40, 41]. For the HADS depression scale, alternative cutoff scores have been proposed for cardiac populations. The cutoff scores that should be used for screening purposes seem to vary depending on the type of cardiac disease [42]. Whether this is the case for the anxiety subscale is still unknown. As compared to the HADS, the STAI has more items and is thus more time consuming to administer and constitutes a greater burden to patients, which is a disadvantage when using it as a screening instrument in clinical practice.

In conclusion, both HADS-A and STAI-S showed good reliability and validity. However, the traditional cutoff scores used to indicate probable clinical levels of anxiety are not equivalent. The HADS-A appears to be more conservative as compared to the STAI-S and will thus produce lower prevalence rates of anxiety. The cutoff scores of the HADS-A seem to vary depending on the type of cardiac disease. Our findings illustrate that studies published to date using the HADS-A and STAI-S to assess anxiety with traditional cutoff scores are not comparable when it comes to prevalence rates. Thus, the prevalence rates should be interpreted in light of the assessment tool used.

### Clinical implications

For clinical practice, it is important to be aware of the fact that a substantial proportion of patients are classified differently using the HADS-A versus the STAI-S. As the traditionally used cutoff score for anxiety on the STAI-S is less conservative as compared to the HADS-A scale, using this scale will result in higher anxiety prevalence rates. This discrepancy has implications for both clinical practice and research. Hence, prior to implementing one of the scales as a screening tool, it is important to decide whether it is more important to preve*nt false positives or false negatives.* To avoid a high number of false positives, the HADS-A scale should be used. By contrast, use of the STAI-S will reduce the number of false negatives. A crosswalk table allows converting total scores from the HADS-A scale to equivalent STAI-S total scores and vice versa, which may be beneficial when comparing patients who completed different questionnaires (e.g., for meta-analyses).

### Limitations

One of the limitations of this study is the fixed order in which the questionnaires were administered, which can be a confounding factor (e.g., carry over effects, fatigue, motivational problems). Second, the crosswalk table provides a useful tool for comparing scores from different scales, but caution should be exercised when applied to individuals in real-life screening settings. Pseudo-HADS-A scores should not be conceived as substitutes for the STAI-S (i.e., should not be seen as the score a person would have should he/she completed the other questionnaire). In addition, the use of generic anxiety measures in the current population might not have tapped sufficiently into the disease-specific anxieties that might be experienced by patients briefly after implantation. Hence, using disease-specific measures to assess anxiety might provide a more accurate reflection of anxiety symptomatology.

### Future research

Future research should investigate whether it is feasible to divide the STAI-S into two scales (present and absent) to comply with the increasing demand for brief measures to reduce the burden to patients and in clinical practice while maintaining prognostic power. In addition, research on the predictive elements of the HADS scale should be considered. A bifactor model for the HADS scale has previously been proposed where anxiety and depression are considered as components of a hierarchical structure that includes a general distress factor [43, 44]. Total scores reflecting the full scope of general distress may be better predictors of poor health outcomes than subscale scores. Furthermore, examining whether different cutoff scores for anxiety should be employed depending on the type of cardiac disease and disease severity is warranted. Finally, results of the crosswalk table seem quite robust (i.e., precise and generalizable), but this needs to be confirmed in future research in other cohorts.
